# The Effects of Guizhi Gancao Decoction on Pressure Overload-Induced Heart Failure and Posttranslational Modifications of Tubulin in Mice

**DOI:** 10.1155/2017/2915247

**Published:** 2017-07-17

**Authors:** Hui-hua Chen, Pei Zhao, Jing Tian, Wei Guo, Ming Xu, Chen Zhang, Rong Lu

**Affiliations:** ^1^Department of Pathology, Shanghai University of Traditional Chinese Medicine, Shanghai 201203, China; ^2^Public Experiment Platform, School of Basic Medical Science, Shanghai University of Traditional Chinese Medicine, Shanghai 201203, China; ^3^Department of Physiology, Shanghai University of Traditional Chinese Medicine, Shanghai 201203, China

## Abstract

Guizhi Gancao Decoction (GGD), a traditional Chinese medical recipe, has been widely used in the treatment of cardiovascular diseases in China for centuries. The present study was carried out to determine whether GGD exerts direct protective effects against pressure overload-induced heart failure. Moreover, we investigated whether GGD affects tubulin expression and posttranslational modifications. We demonstrated that GGD ameliorated TAC caused cardiac hypertrophy by gravimetric and echocardiography analysis in C57BL/6 mice. We found that GGD abrogated TAC-induced myocardium fibrosis by Masson's staining and collagen volume fraction (CVF) analysis. By using pressure-volume hemodynamic measurements, we found that GGD prevented TAC-induced cardiac systolic and diastolic dysfunction. Immunoblotting and immunofluorescent analysis revealed that GGD abrogated TAC-induced detyrosination and acetylation abnormalities on microtubules. Our present study demonstrated potential therapeutic effects of GGD against pressure overload-induced heart failure.

## 1. Introduction

Pressure overload imposes hemodynamic burden on the left ventricle (LV), which initiates a series of events leading to ventricular remodeling, including hypertrophy (LVH) and fibrosis, and eventually heart failure. Although in the early stages LVH may seem to be an adaptive phenomenon, it has been recognized as an independent risk factor for cardiovascular morbidity and mortality, including heart failure [1]. Pressure overload-induced LVH is accompanied by the accumulation of collagen in the extracellular matrix, which alters myocardial stiffness and consequently affects cardiac function [2]. Recently, emerging evidences demonstrated that hypertrophied heart is accompanied by proliferating of cardiac microtubules and altered the posttranslational modifications (PTMs) on tubulin, such as detyrosination and acetylation of tubulin [3].

Guizhi Gancao Decoction (GGD), which consists of Guizhi (*Cinnamomum cassia *Presl.) and Gancao (*Glycyrrhiza uralensis*), was originally described in Han dynasty. It has been widely used for therapy against cardiovascular diseases in China for thousands years. Modern pharmacological studies have revealed serval bioactive constituents of GGD that may have cardioprotective effects. Yang et al. reported that Cinnmaldehyde attenuates aortic banding induced cardiac hypertrophy and fibrosis probably via blocking ERK signaling pathway [4]. Du et al. demonstrated that glycyrrhizin and some of its derivatives treat heart failure by inhibiting High-Mobility Group Box 1 [5]. Parisella et al. have revealed that glycyrrhizin has positive inotropic and lusitropic effects which were negatively affected by glycyrrhetinic acid. They also found that glycyrrhizin acts through the endothelin receptor type A/phospholipase C axis, while glycyrrhetinic acid acts through endothelin receptor type B/Akt/nitric oxide synthase/nitric oxide axis [6]. However, the underling mechanisms of the potential therapeutic effects of GGD on heart failure remain elusive. The present research was designed to document the beneficial effects of GGD on pressure overload-induced heart failure on mice and to investigate the role of GGD on the expression and posttranslational modifications of *α*-tubulin.

## 2. Materials and Methods

### 2.1. Preparation of Guizhi Gancao Decoction (GGD)

GGD is composed of two Chinese herbs, which are Guizhi (*Cinnamomum cassia *Presl., 12 g) and Gancao (*Glycyrrhiza uralensis*, 6 g). Both herbs were supplied by the Shanghai Kangqiao Chinese Medicine Tablet Co., Ltd (Shanghai, China). Five times of the normal dosage for adult humans was defined as the dosage of GGD for mice. Thus, mice in GGD groups were given 1.5 g/kg/day of GGD. The dosage of GGD represents the dry weight of the raw herbs used to produce decoction.

### 2.2. Experimental Animals and TAC Surgery

All animal procedures described in this study were approved by the Animal Care and Use Committee of Shanghai University of Traditional Chinese Medicine. Adult male C57BL/6 mice (weighing 18–22 g) were purchased from the Shanghai SLAC Laboratory Animal Center (Shanghai, China). The animals were housed at room temperature under a 12 h light/dark cycle with free access to water and standard diet. The transverse aortic constriction (TAC) surgery was used to generate pressure overload-induced cardiac hypertrophy and heart failure as previously described [7]. Briefly, mice were anesthetized with isoflurane (Inspira-ASV, Harvard Apparatus, Holliston, MA, USA) and placed supine on a warm electric pad (World Precision Instruments, Inc., Sarasota, FL, USA). The transverse aortic arch was ligated between the innominate artery and the left carotid artery with a 27-gauge intravenous catheter using a 6-0 nylon suture. The catheter was removed after the ligation. The animals in SHAM group underwent all operation procedures except the ligation. Animals subjected to TAC were treated with GGD (1.5 g/kg), telmisartan (Tel, 8 mg/kg, as a positive control), or tap water (as a vehicle control) by oral gavage for 4 weeks. Telmisartan, an angiotensin II receptor blocker, has been shown to suppress cardiac hypertrophy and collagen deposition induced by pressure overload both in patients and in animal models [8, 9].

### 2.3. Echocardiography Analysis

The animals were anesthetized with 2.5% isoflurane in 95% oxygen and 5% carbon dioxide. Animals were placed on controlled heating pads, while the core temperature was maintained at 37°C. Left ventricular function was evaluated by echocardiography using a high-resolution small animal imaging system (Vevo2100, VisualSonics Inc., Toronto, Canada), and the animal situated in the supine position on a warming platform. Two-dimensional and M-mode echocardiographic images of long and short axis were recorded. On two-dimensional recordings of short axis at the mid-papillary muscle level, left ventricle posterior wall thickness (LVPW), interventricular septal wall thickness (IVSD), and left ventricle diameters (LVID) in diastole and systole were measured. Left ventricle ejection fraction (LVEF) and fractional shortening (LVFS) were calculated (VisualSonics software).

### 2.4. Hemodynamics Measurement

Cardiac function was measured by pressure-volume loop as previously described [10]. Briefly, animals were anesthetized with isoflurane, fixed, and intubated/respirated. After chest opening, a 1.2 F pressure-volume (PV) conductance catheter (Scisence, Ontario, Canada) was inserted into the left ventricle via an apex approach and data were recorded and analyzed using a LabScribe2 software (iWorx, Dover, NH, USA). After stabilization for 15 min, 10–20 steady-state pressure-volume loops were collected. The measured steady-state hemodynamic parameters include derivative of pressure (*dp*/*dt*max, *dp*/*dt*min) and isovolumic relaxation constant (Tau). After that, the inferior vena cava was transiently occluded for 3 seconds. During this period, the slope of the end-systolic pressure-volume relationship (ESPVR), preload recruitable stroke work (PRSW), and the slope of the end-diastolic pressure-volume relationship (EDPVR) were measured.

### 2.5. H&E Staining and Masson's Trichrome Staining

The mice were all sacrificed, and the body weight, heart weight, and tibia length were determined. The heart tissues were fixed in 4% paraformaldehyde overnight at 4°C, rinsed, and transferred to PBS followed by paraffin embedding. Sections were stained with hematoxylin and eosin. After conventional deparaffin of the paraffin sections, Masson's trichrome staining was performed to evaluate myocardial fibrosis. Myocardial cells were stained red and collagenous fibers stained blue. The collagen deposition was quantitatively analyzed by collagen volume fraction (CVF) via Metamorph image process (Universal Imaging Corp, USA). The calculation formula of CVF in each view of the slice is CVF = collagen  area/total  area × 100%.

### 2.6. Immunofluorescent Staining

For immunohistochemistry assay, the heart tissue was frozen in OCT in liquid nitrogen and sectioned at 0.5 *μ*m. The sections were incubated with a primary antibody against detyrosinated *α*-tubulin (Abcam, Cambridge, CB, UK), followed by Alexa Fluor 568 (red, Fisher Scientific, Waltham, MA, USA) and then the sections were stained with DAPI (Cell Signaling Technology, Danvers, MA, USA). Images were obtained at 568 nm excitation by a Carl Zeiss LSM800 confocal microscope (Carl Zeiss Microcopy GmbH, Jena, Germany).

### 2.7. Western Blot Analysis

Protein lysates from heart tissues were loaded on and separated by a 12% SDS-PAGE and then transferred to PVDF membranes (EMD Millipore, MA, USA). The membranes were probed overnight with primary antibodies at 4°C. The primary antibodies were anti-detyrosinated *α*-tubulin (Abcam, Cambridge, CB, UK), anti-acetylated *α*-tubulin (Abcam, Cambridge, CB, UK), anti-*α*-tubulin (Abcam, Cambridge, CB, UK), anti-tubulin tyrosine ligase (Proteintech, Rosemont, IL, USA), anti-HDAC6 (Abcam, Cambridge, CB, UK), and anti-GAPDH (Abcam, Cambridge, CB, UK). Membranes were incubated with appropriate secondary antibodies for 2 h at room temperature. Membranes were imaged using Odyssey infrared fluorescence imager (LI-COR, Lincoln, NE, USA). Some of the signals were detected using Immobilon Western Chemiluminescent HRP Substrate (EMD Millipore, MA, USA).

### 2.8. Statistical Analysis

All values were analyzed with SPSS21.0 and expressed as means ± SEM. Multiple comparisons between groups were examined using one-way analysis of variance (ANOVA) followed by Tukey's post hoc analysis, and *P* < 0.05 were considered statistically significant.

## 3. Results

### 3.1. Effects of GGD on Cardiac Hypertrophy

**Figure 1:**
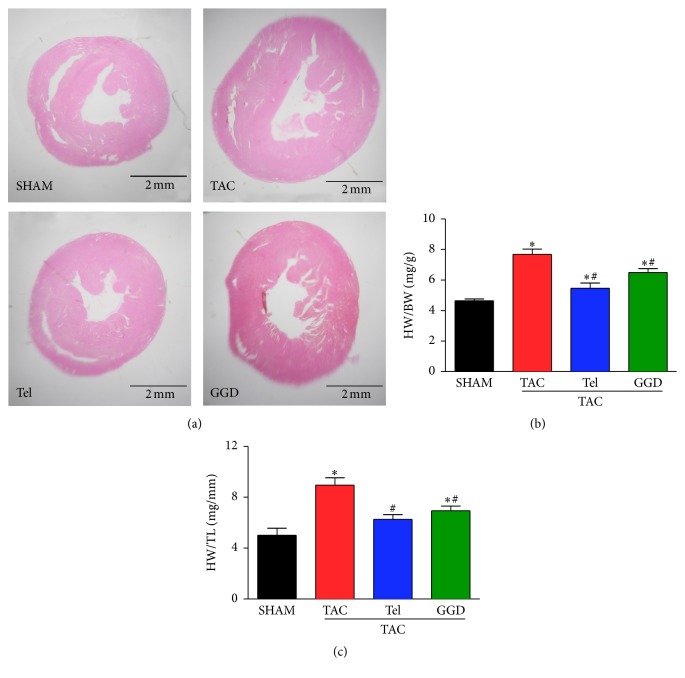
Effects of GGD on cardiac hypertrophy. (a) Cardiac images: hearts were sectioned at papillary muscle level, H&E staining, ×8. (b) Quantification of heart weight-to-body weight ratio. (c) Quantification of heart weight-to-tibial length ratio. The data are expressed as Mean ± SEM. ^*∗*^*P* < 0.05 versus SHAM; ^#^*P* < 0.05 versus TAC. HW: heart weight; BW: body weight; TL: tibial length; TAC: transverse aorta constriction; Tel: telmisartan; GGD: Guizhi Gancao Decoction.

As shown in Figures 1(b) and 1(c), TAC operation significantly increased the ratio of heart weight (HW)/body weight (BW) and the ratio of HW to tibia length (TL) as well. GGD treatment partially but significantly reduced the increase in HW/BW ratio and HW/TL ratio. Meanwhile the positive control medicine Tel completely prevented the increase in HW/TL ratio and partially but significantly reduced the increase in HW/BW ratio. These parameters are consisted with the HE stained histology sections (Figure 1(a)), which showed that GGD or Tel administration blocked TAC caused increase in LV wall thickness.

### 3.2. Effects of GGD on Myocardium Fibrosis

**Figure 2:**
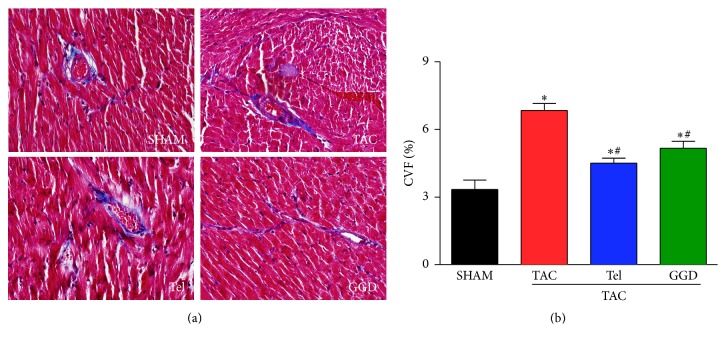
Effects of GGD on myocardium fibrosis. (a) Cardiac images: Masson's trichrome staining, ×400. (b) Quantification of CVF. The data are expressed as Mean ± SEM. ^*∗*^*P* < 0.05 versus SHAM; ^#^*P* < 0.05 versus TAC. CVF: collagen volume fraction; TAC: transverse aorta constriction; Tel: telmisartan; GGD: Guizhi Gancao Decoction.

Using Masson's staining, we found that animals subjected to TAC operation exhibited more severe myocardium fibrosis compared with controls. The collagen mostly deposited in perimysium and perivascular region. Animals subjected to GGD or Tel treatment demonstrated almost normal myocardium interstitium (Figure 2(a)). Moreover, mice subjected to TAC had significantly increased value of CVF which was significantly attenuated by GGD or Tel treatment (Figure 2(b)).

### 3.3. Effects of GGD on Echocardiographic Parameters

**Figure 3:**
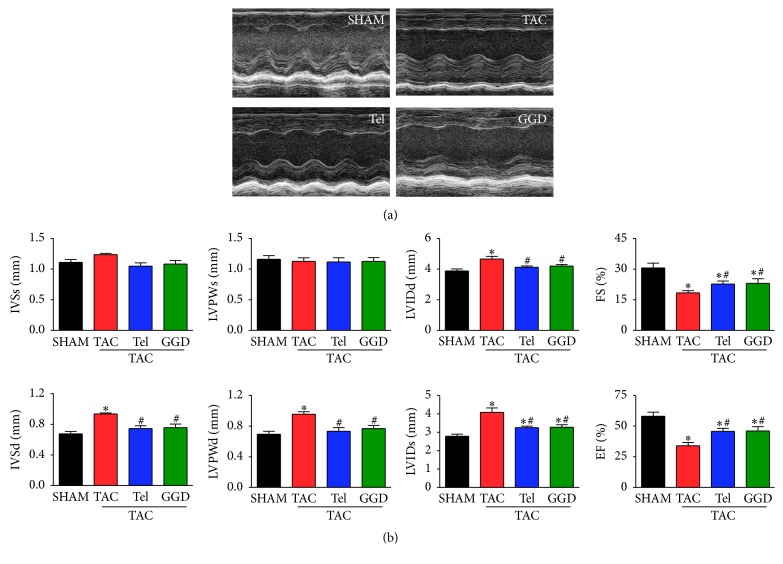
Effects of GGD on echocardiographic parameters. (a) Representative tracing of echocardiography. (b) Quantification of echocardiography parameters. The data are expressed as Mean ± SEM. ^*∗*^*P* < 0.05 versus SHAM; ^#^*P* < 0.05 versus TAC. LVPW: ventricle posterior wall thickness; IVSD: interventricular septal wall thickness; LVID: left ventricle diameters; LVEF: left ventricle ejection fraction; LVFS: fractional shortening; TAC: transverse aorta constriction; Tel: telmisartan; GGD: Guizhi Gancao Decoction.

The morphological and functional echocardiographic parameters are shown in Figure 3. The LV wall thickness values, including IVS and LVPW, were significantly increased in mice subjected to TAC in diastolic phase. GGD or Tel treatment can significantly reduce the values, although they are still significantly greater than SHAM group. However, in systolic phase these values are unaffected by TAC with or without GGD or Tel treatment.

Dilation of LV in mice subjected to TAC was observed as the values of LVID were significantly increased both in diastolic and in systolic phase compared with SHAM operated mice. TAC-induced increase in LVIDs was completely blocked by GGD or Tel, whereas TAC-induced increase in LVIDs was significantly reduced but still significantly different from SHAM operated mice.

Echocardiography also showed TAC produced LV systolic dysfunction which was blocked by GGD treatment. As shown in Figure 3, LVEF and LVFS in mice subjected to TAC were significantly lower than SHAM operated mice, which can be partially but significantly prevented by GGD or Tel treatment.

### 3.4. Effects of GGD on Hemodynamic Parameters

Baseline hemodynamic data showed that TAC with the presence or absence of GGD or Tel did not alter the *dp*/*dt*max value. However, TAC significantly reduced the value of *dp*/*dt*min, which was completely prevented by GGD or Tel treatment. TAC also significantly prolonged Tau which was completely prevented by GGD, but not by Tel.

**Figure 4:**
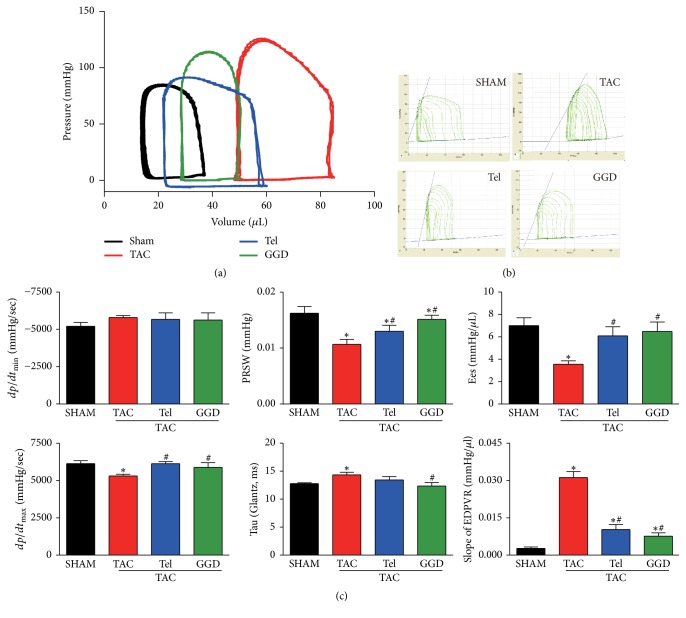
Effects of GGD on hemodynamic parameters. (a) Representative steady-state pressure-volume loops. (b) Representative pressure-volume loops during vena cava occlusion. (c) Quantification of hemodynamic parameters. The data are expressed as Mean ± SEM. ^*∗*^*P* < 0.05 versus SHAM; ^#^*P* < 0.05 versus TAC. *dp*/*dt*: derivative of pressure; Tau: isovolumic relaxation constant; PRSW: preload recruitable stroke work; Ees: the slope of ESPVR (the end-systolic pressure-volume relationship); EDPVR: the slope of the end-diastolic pressure-volume relationship; TAC: transverse aorta constriction; Tel: telmisartan; GGD: Guizhi Gancao Decoction.

Recording pressure-volume loop data during transient inferior vena cava occlusion allows us to analyze preload-independent LV function parameters. As shown in Figures 4(b) and 4(c), TAC operation significantly decreased the slope of PRSW which was partially but significantly increased by GGD or Tel treatment. Similarly, Ees (the slope of ESPVR) was significantly decreased by TAC operation. This response was completely prevented by GGD or Tel treatment, whereas the slope of EDPVR was significantly increased by TAC operation which was partially but significantly prevented by GGD or Tel treatment.

### 3.5. Effects of GGD on Cardiac *α*-Tubulin Expression and Posttranslational Modifications

**Figure 5:**
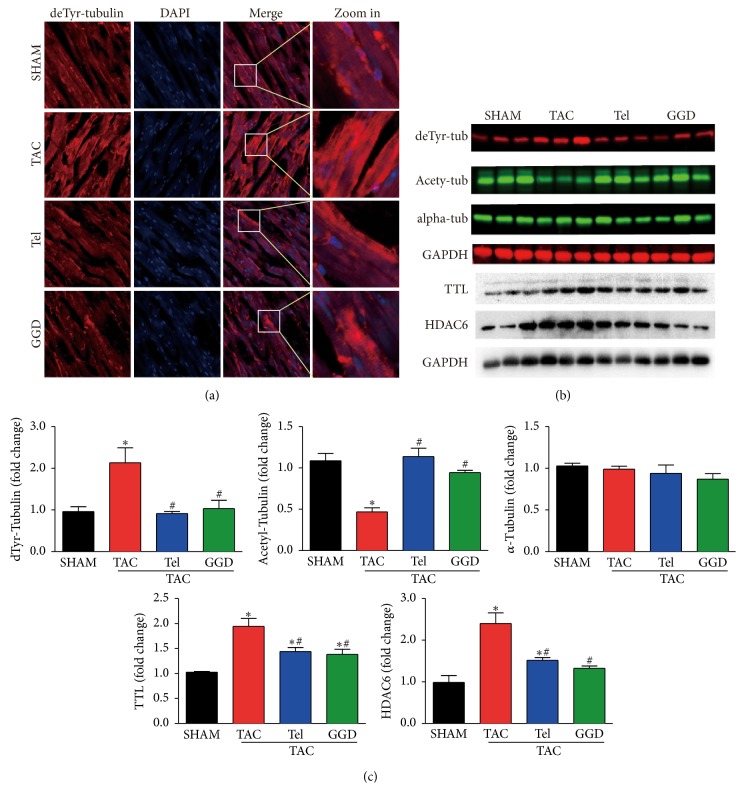
Effects of GGD on cardiac *α*-tubulin expression and posttranslational modifications. (a) Representative immunofluorescence images with anti-detyrosinated tubulin (red) and DAPI (blue). ×400. (b) SDS-PAGE and western blot bands. (c) Quantification of (b). The data are expressed as Mean ± SEM. ^*∗*^*P* < 0.05 versus SHAM; ^#^*P* < 0.05 versus TAC. TTL: tubulin tyrosine ligase; HDAC6: histone deacetylase 6; TAC: transverse aorta constriction; Tel: telmisartan; GGD: Guizhi Gancao Decoction.

As shown in Figures 5(b) and 5(c), *α*-tubulin expression was unaffected by TAC operation with or without the presence of GGD or Tel. Meanwhile, detyrosinated *α*-tubulin was significantly increased by TAC operation, which was normalized by GGD or Tel treatment. This result wa consisted with immunofluorescent data shown in Figure 5(a). Acetylated *α*-tubulin was significantly decreased in animals subjected to TAC operation compared with SHAM operation, which was completely prevented by GGD or Tel treatment. We then examined two crucial enzymes for posttranslational modifications of *α*-tubulin, namely, TTL and HDAC6. As shown in Figures 5(b) and 5(c), TTL expression was significantly increased by TAC operation which was partially but significantly decreased by GGD or Tel treatment. In addition, TAC increased HDAC6 expression, which was completely abrogated by GGD and partially but significantly increased by Tel.

## 4. Discussion

In this study, we demonstrate GGD attenuated TAC-induced cardiac hypertrophy and myocardium fibrosis. We also show that GGD can prevent cardiac function decline caused by TAC operation. Moreover, our study shows that GGD prevented TAC caused abnormality in *α*-tubulin posttranslational modifications.

A hallmark feature of ventricular remodeling is the deposition of excessive extracellular matrix proteins, mostly the collagens. Cardiomyocytes death and other pathological stimuli, including chronic pressure or volume overload, will trigger profibrotic pathways. Fibrosis arising from cardiomyocyte death and replaced with scar is so-called replacement fibrosis. In contrast, fibrosis arising during mechanic overload is reactive fibrosis, where collagen deposition mainly happened in perivascular or interstitial regions, as shown in Figure 2(a) [11]. Reactive fibrosis causes decreased compliance, contributing to diastolic or even systolic dysfunction [12]. GGD that prevented TAC-induced myocardium fibrosis may contribute to the beneficial effects of cardiac function.

Pathological cardiac hypertrophy is another vital and independent predictor of heart failure [13]. According to the results of echocardiography and gravimetric analysis, LV wall thickness and heart weight index values were significantly increased in animals subjected to TAC compared with controls. TAC-induced cardiac hypertrophy was prevented by GGD. We also observed a significant cardiac dysfunction caused by TAC operation, as indicated by the enlarged left ventricular chamber size and marked impairment in cardiac contractile function (EF and FS), which can be partially attenuated by GGD.

LV pressure-volume loop analysis and load-independent functional indexes measurement confirmed that GGD prevented TAC-induced hemodynamic abnormalities. It is well recognized that *dP*/*dt*max and echocardiographic parameters, including EF and FS, are largely depending on loading conditions, especially the preload and heart rate [14]. The slope of ESPVR (Ees) and PRSW, on the other hand, have been proposed as load-independent indexes of ventricular contractility. According to present results, GGD prevented TAC-induced increase in Ees and PRSW, indicating that GGD administration alleviated the impairment of LV contractile state. GGD treatment also blocked TAC-induced steeper slope of EDPVR and prolonged Tau, which suggested that GGD abrogated TAC-induced diastolic dysfunction.

Microtubules are the largest cytoskeletal components, which are assembled from heterodimers of *α*-tubulin and *β*-tubulin. Soluble *α*-tubulin-*β*-tubulin dimers polymerize into microtubules in the presence of GTP. Microtubules are highly dynamic, undergoing cycles of polymerization and depolymerization [15]. It has been reported that excessive microtubule polymerization was involved in contractile dysfunction in pressure overload-induced heart failure [16, 17]. The dynamic equilibrium of microtubule is regulated by enzymes and binding proteins [18]. There is emerging evidence that the posttranslational modifications on microtubules, such as detyrosination and acetylation, are crucial controllers of microtubule properties and functions [19]. Detyrosination is the removal of the C-terminal tyrosine from *α*-tubulin, and it is estimated that approximately 60% of *α*-tubulin is undergoing detyrosination [20]. It has been demonstrated that the amount of detyrosinated tubulin was increased in animal models and patients with heart failure [21, 22]. In this study, we found that GGD blocked the increase in detyrosinated microtubules induced by TAC. To date, the carboxypeptidase which catalyzes detyrosination is still unknown, while the readdition of tyrosine residue is catalyzed by the tubulin tyrosine ligase (TTL) [23]. The detyrosination-tyrosination cycle is initiated by the detyrosination [24]. This suggested that the overexpression of TTL in animals subjected to TAC and the ability of GGD to inhibit TTL expression in this study might be secondary to the changes of detyrosination level of tubulin. Acetylation of Lys40 on *α*-tubulin is another PTM that happened on the microtubule polymer [25]. It has been suggested that the major *α*-tubulin acetyltransfere is *α*TAT1, although it remains controversial [26]. However, the reverse reaction, deacetylation of *α*-tubulin, is known to be catalyzed by histone deacetylase 6 (HDAC6) and SIRT2 [27]. Inhibition of HDAC6 activity increases tubulin acetylation and protects against cardiomyopathy [28]. Our data showed that GGD inhibited HDAC6 and increased tubulin acetylation, which may have beneficial effects on heart failure. These effects are consisted with one report that cinnamic acid, one of the major constituents of* Cinnamomum cassia* Presl., inhibited HDAC activity [29].

In conclusion, this study demonstrates that GGD has cardioprotective activities against TAC-induced heart failure; these therapeutic effects might correlate with modulation of PTMs on microtubules. Our study provides experimental evidences for the application of GGD in the treatment of heart failure.
